# Mechanical Characterization and Constitutive Modeling of Human Trachea: Age and Gender Dependency

**DOI:** 10.3390/ma9060456

**Published:** 2016-06-08

**Authors:** Farzaneh Safshekan, Mohammad Tafazzoli-Shadpour, Majid Abdouss, Mohammad B. Shadmehr

**Affiliations:** 1 Faculty of Biomedical Engineering, Amirkabir University of Technology, 424 Hafez Ave, Tehran 1587-4413, Iran; f.safshekan@aut.ac.ir; 2 Chemistry Department, Amirkabir University of Technology, Tehran 1587-4413, Iran; phdabdouss44@aut.ac.ir; 3 Tracheal Diseases Research Center, National Research Institute of Tuberculosis and Lung Diseases (NRITLD), Shahid Beheshti University of Medical Sciences, Tehran 1956944413, Iran; mbshadmehr@sbmu.ac.ir

**Keywords:** trachea constituents, mechanical behavior, hyperelastic model, aging, cartilage, connective tissue, smooth muscle

## Abstract

Tracheal disorders can usually reduce the free lumen diameter or wall stiffness, and hence limit airflow. Trachea tissue engineering seems a promising treatment for such disorders. The required mechanical compatibility of the prepared scaffold with native trachea necessitates investigation of the mechanical behavior of the human trachea. This study aimed at mechanical characterization of human tracheas and comparing the results based on age and gender. After isolating 30 human tracheas, samples of tracheal cartilage, smooth muscle, and connective tissue were subjected to uniaxial tension to obtain force-displacement curves and calculate stress-stretch data. Among several models, the Yeoh and Mooney-Rivlin hyperelastic functions were best able to describe hyperelastic behavior of all three tracheal components. The mean value of the elastic modulus of human tracheal cartilage was calculated to be 16.92 ± 8.76 MPa. An overall tracheal stiffening with age was observed, with the most considerable difference in the case of cartilage. Consistently, we noticed some histological alterations in cartilage and connective tissue with aging, which may play a role in age-related tracheal stiffening. No considerable effect of gender on the mechanical behavior of tracheal components was observed. The results of this study can be applied in the design and fabrication of trachea tissue engineering scaffolds.

## 1. Introduction

The trachea is a hollow semi-cylindrical organ which not only acts as an airway but also contributes to the drainage of secretions of bronchi and bronchioles. The human trachea is composed of 18 to 22 consecutive C-shaped cartilaginous rings which are incomplete in the posterior region of the trachea and completed by the trachealis muscle, also known as smooth muscle tissue. The narrow spaces between cartilaginous rings are filled with connective tissue [1,2]. Other soft connective tissues of the trachea include the adventitia membrane as the outermost layer of the trachea, along with mucosa and submucosa membranes covering its inner surface [3].

There is a close relationship between the mechanical performance of the trachea and its physiological function during respiration. The stiff cartilage components keep the trachea lumen open and inhibit its collapse during negative respiratory pressures, and hence prevent the air flow limitation. On the other hand, the deformation of smooth muscle tissue exerts tensile and bending stresses on the cartilaginous rings and modulates the lumen diameter during respiration [4].

Tracheas can be exposed to different disorders, such as inflammatory or traumatic conditions, tumors, and infections [5], that can usually result in stenosis (reduction in the free lumen diameter) or malacia (reduction in the wall stiffness), both of which can limit the air flow and cause breathing problems [6].

To treat most severe tracheal disorders, after resection of the injured part of the trachea, the ends of the remaining parts are joined together. This widely-used procedure, which is known as resection-anastomosis surgery [7], is limited with the allowable length of trachea for resection and can result in a stretched and less deformable airway during respiration. Since most soft tissues including smooth muscle and connective tissue between the cartilaginous rings follow stiffening behavior (incremental Young’s modulus with increasing strain), any stretch as it happens during the surgery results in a stiffer (and less deformable) trachea compared to pre-surgery conditions, thereby causing some breathing difficulties. As alternative treatments for larger tracheal injuries, trachea stents or implants are used [8], but they are also associated with some problems such as displacement, material degradation or failure, infection, formation of granulated tissue, and stenosis, as well as lack of vascularization [9]. As such methods have not been found to be practical, tissue engineering approaches using living cells and scaffolds to generate tissues seem promising. The main challenge in trachea tissue engineering is to obtain a scaffold with mechanical properties and function compatible with those of the native trachea, so as to be able to properly function after transplantation [10].

To obtain the required mechanical properties of tracheal tissue engineered constructs, it is first necessary to characterize mechanical behavior of the native trachea. Pioneer efforts on determining mechanical properties of trachea had focused on experimental measurements to obtain “tube law”, (*i.e.*, the pressure-cross sectional area relationships) for excised or intact trachea (see [11] for a full review). However, in recent years, mechanical properties of single tracheal components such as cartilage, smooth muscle, *etc.* have attracted scientific interests as they allow detailed analytical examination and modeling of tracheas. In this regard, some aspects of material behavior such as isotropy *vs.* anisotropy (*i.e.*, exhibiting the same or different mechanical properties in different directions, respectively), compressibility *vs.* incompressibility (change or no change in the volume of the material in response to the applied pressure), and linear *vs.* non-linear elastic behavior (with the stress-stretch relationship being a straight line or a non-linear relationship derived from a strain energy potential function) have been used to describe the mechanical behavior of such tissues.

In previous studies, tracheal cartilage has been assumed as an incompressible [2,12,13,14,15] or compressible [16] isotropic material, with a linear [4,16,17] or non-linear elastic behavior [2,6,14,15,18]. Tracheal cartilage has been also considered as a transversely isotropic tissue [12,13]. Tracheal smooth muscle has been previously considered as an incompressible [2,13,15] or compressible [16] material, as being either an isotropic [2,15,16] or a transversely isotropic [6,13,14,18] hyperelastic tissue. In the few studies on mechanical properties of tracheal connective tissue, it has been considered as an isotropic and hyperelastic material [2,15,16].

Among different investigations, only in a few studies on the trachea of lambs [2,16], leatherback sea turtles [15], and goats [19], all the three tracheal components including cartilage, smooth muscle, and connective tissue have been mechanically described, and to the best of our knowledge, such a complete investigation to mechanically characterize human trachea using a sufficient number of samples and taking all the three main components into account has not been carried out yet.

Age-related alterations in biological tissues make it necessary to mechanically study various subjects with a wide range of ages, so as to obtain reliable conclusions, independent of the age factor [14]. On the other hand, comparing the mechanical properties of tissues between young and old subjects, or in terms of gender, would be of great significance and can broaden the scope for fabrication of tissue engineered scaffolds or similar treatments.

Hence the aim of this work was to determine the elastic properties of major components of human tracheas and compare the results based on age and gender.

## 2. Results

### 2.1. Stress-Stretch Curves

**Figure 1:**
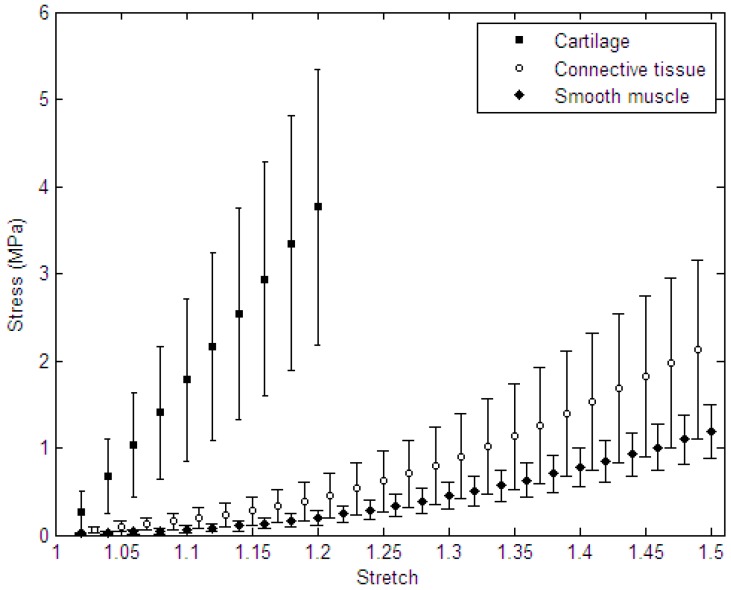
The average Cauchy stress-stretch data for cartilage, smooth muscle, and connective tissue samples (with average initial lengths (*L*_0_) of 4.13 ± 1.81 mm, 6.37 ± 1.91, and 1.60 ± 0.33 mm, respectively). Data were expressed as mean ± STD.

The force-displacement curves obtained from uniaxial tensile tests were used to calculate Cauchy stress-stretch data using Equations (2) and (3). The average stress-stretch curves corresponding to the three tracheal components (*i.e.*, cartilage, smooth muscle, and connective tissue) are shown in Figure 1. It is notable that to include all samples in the averaging process, such calculations were performed until a stretch (*λ*) of 1.2 (*i.e.*, 20% strain) was recorded for cartilage, and 1.5 for both smooth muscle and connective tissue, which are well higher than the physiologic deformation of tracheal constituents. Based on the results, cartilage tissue showed the highest stiffness among the three tracheal components, and its mechanical behavior was well-fitted by the linear model (*R*^2^ = 0.999) with the average Young’s modulus of 16.92 ± 8.76 MPa. The stress-stretch curves of smooth muscle and connective tissue were well indicative of the “stiffening” behavior of such soft tissues.

### 2.2. Hyperelastic Modelling

For each tracheal component, first the average stress-stretch curve was obtained and then different hyperelastic models were used to fit such experimental data. According to the goodness of fits, the best model for describing the mechanical behavior of each tissue was selected. The fitting goodness (coefficient of determination, *R*^2^) of different hyperelastic models for describing each tissue are listed in Table 1.

**Figure 2:**
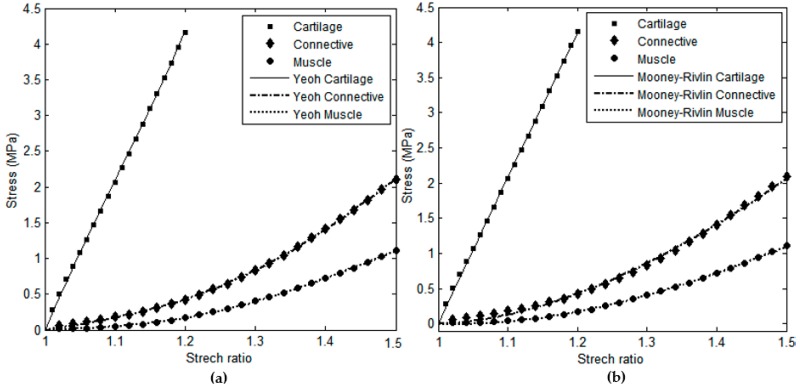
(**a**) Yeoh and (**b**) Mooney-Rivlin models fitted to the experimental stress-stretch averaged data for cartilage, connective tissue, and smooth muscle.

Based on the results, for each tissue multiple models were fitted to the experimental data with *R*^2^ values close to 1. This was most evident in the case of tracheal cartilage, which exhibited an almost linear stress-stretch behavior, as previously stated, and could be well-fitted by most of the studied hyperelastic models. The 3-term Yeoh and Mooney-Rivlin models were chosen as the two best-fitting hyperelastic models for all the three tracheal components as illustrated in Figure 2. The corresponding coefficients of the models for each tissue along with the fitting goodness are listed in Table 2. As it can be seen in Figure 2, both models were well able to capture the stress-stretch behavior of tracheal cartilage, connective tissue, and smooth muscle.

### 2.3. The Effects of Age and Gender on the Mechanical Behavior

**Figure 3:**
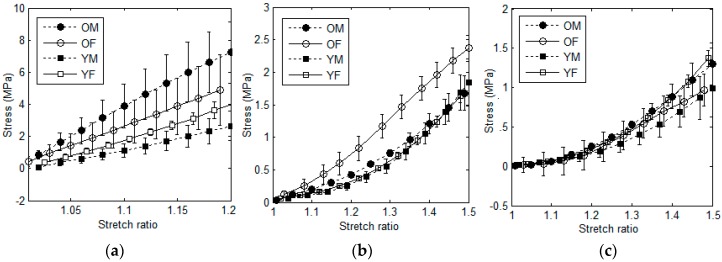
The average curves of experimental stress-stretch data for tracheal tissues of young and old samples related to male and female subjects: (**a**) cartilage; (**b**) connective tissue; (**c**) smooth muscle.

The resulting stress-stretch curves obtained for each tracheal tissue *(i.e.*, cartilage, smooth muscle, and connective tissue) were averaged and the final mean curves for young and aged samples corresponding to male and female subjects were obtained. Results are illustrated in Figure 3.

**Figure 4:**
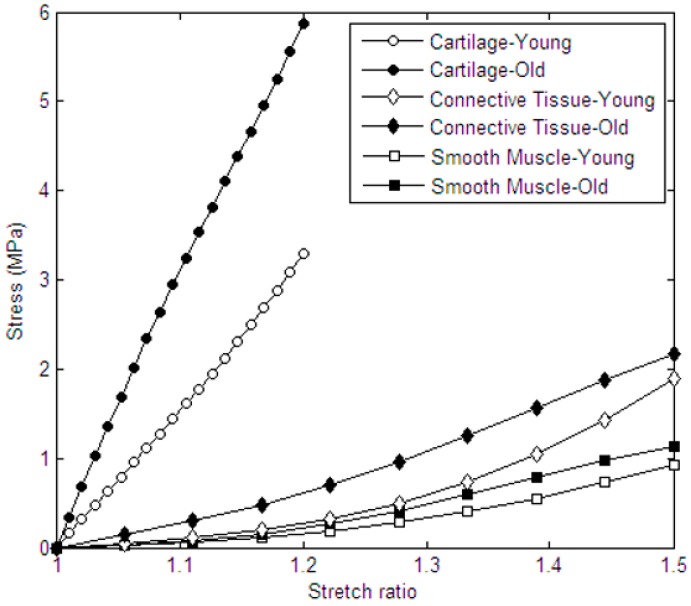
Yeoh model fitted to the average stress-stretch curves corresponding to tracheal samples of young and old subjects.

As it can be seen in Figure 3, in the case of all three tissues, older samples were generally stiffer than the young ones. For instance, for both male and female subjects, tracheal cartilages of the old samples were stiffer than those of the younger ones. The corresponding elastic moduli were 13.30 ± 5.72 MPa and 20.71 ± 10.17 MPa for young and old samples, respectively. Such stiffer behavior of older samples was observed in the case of both connective tissue (Figure 3b) and smooth muscle (Figure 3c), although with lesser effect. To better compare the data corresponding to young and old samples regardless of gender, the Yeoh model was fitted to such experimental groups as illustrated in Figure 4. As it can be observed, cartilage was the most age-sensitive tracheal tissue, and the stiffness values for old samples were significantly higher than those of the young ones (*p*-value < 0.05). In the case of both connective tissue and smooth muscle, while the obtained curves were indicative of a slightly stiffer behavior of old samples, no statistically significant difference between old and young subjects was observed; as for these tissues, statistical analysis revealed *p*-values higher than 0.05. However, regarding such *p*-values, one can consider smooth muscle as the least age-sensitive component with the highest *p*-value obtained during age-based comparison of the mechanical properties. In summary, the two-factor ANOVA analysis revealed a significant effect of age factor only on the behavior of tracheal cartilage (and not in the case of connective tissue and smooth muscle). In addition to this, there was no significant effect of gender on the results. No significant interaction between age and gender factors was observed as well.

### 2.4. Histological Study

**Figure 5:**
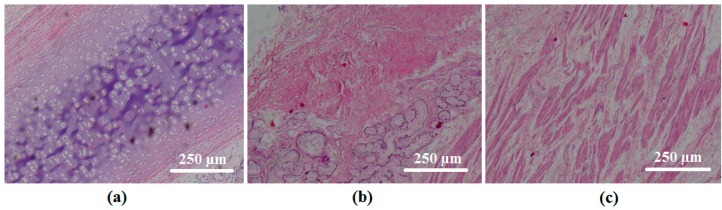
Haematoxylin and eosin (HE) staining of tracheal (**a**) cartilage (40×), indicative of the presence of chondrocytes as round white cells in the homogenized cartilaginous matrix in purple; (**b**) connective tissue (40×), as a loose and irregular tissue including tracheal glands as round regions; and (**c**) smooth muscle, including striations as red elongated fibrous regions (40×).

Observation of the microstructure of stained tissues revealed the fibrous nature of tracheal cartilage, smooth muscle, and connective tissue. Figure 5 illustrates representative images of such tracheal components.

**Figure 6:**
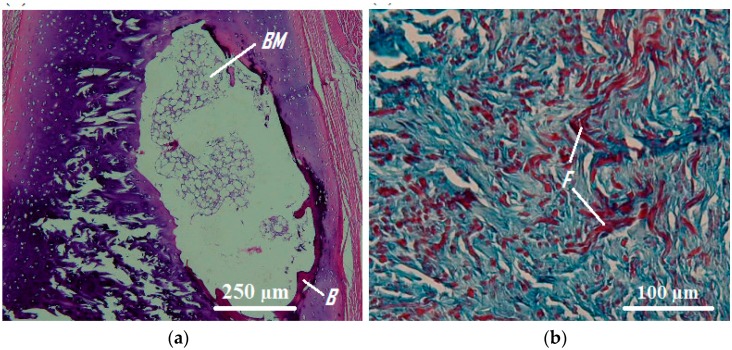
(**a**) Ossified tracheal cartilage of a 56-year old female subject in which the big bright region (*BM*) is bone marrow, surrounded by bone (*B*) (HE staining, 40×) and (**b**) tracheal connective tissue of an old subject (Masson’s trichrome, 100×), where red regions (*F*) demonstrate the fibrosis tissue.

As the mechanical properties of cartilage and connective tissue were more sensitive to the age of samples (Figure 4), the age dependency of microstructure of these two tracheal components was further examined. As illustrated in Figure 6a, ossification of the tracheal cartilage with aging was evident. In this picture, the big bright region (*BM*) is indicative of the formed bone marrow surrounded by small bone regions (*B*) in red. In the case of connective tissue, as shown in Figure 6b the formation of fibrosis tissue with aging could be observed. Here, red regions (*F*) are indicative of fibrosis tissue, the existence of which can stiffen the tissue.

## 3. Discussion

In the present study, the human trachea was mechanically characterized. Compared to previous studies on a limited number of subjects, here the largest population of subjects was studied, allowing investigation of age- and gender-related alterations in mechanical properties. Since a trachea is composed of multiple tissues, to mechanically assess this tissue one should consider it as a composite material and examine the mechanical properties of its individual elements.

Consistent with a previous study [2], here we mechanically characterized cartilage, smooth muscle, and the connective tissue between consecutive cartilaginous rings as the main structural components of a trachea. The mechanical properties of other soft tissues such as adventitia, mucosa, and submucosa membranes were not investigated due to their relatively low contribution to the mechanical performance of tracheas [3,20].

Cartilage, smooth muscle, and connective tissue were all assumed as incompressible materials due to their high water content and fibrous structure [21], which is consistent with previous investigations [2,12,13,14,15]. We considered such tracheal components as isotropic and hyperelastic materials, all of which could be best-fitted by the Yeoh and Mooney-Rivlin models. Considering tracheal cartilage as a non-linear elastic material, Neo-Hookean [6,14,18], Marlow [2], or Mooney-Rivlin [15] strain energy functions with isotropy assumption and Fung strain energy function [12,13] assuming transverse isotropy had been previously used to describe its mechanical behavior.

In this study, up to a stretch (*λ*) of 1.2, the stress-stretch behavior of cartilage could also be well-fitted by the linear model, which is consistent with previous studies assuming a completely linear elastic behavior for this tissue [4,16,17], or at least until a certain strain level [22]. Such an extension level (*i.e.*, a stretch of 1.2) fully covers the physiologic deformation range of tracheal cartilage which is normally under the stretch of 1.05 [14,23].

According to our results, the elastic modulus of human tracheal cartilage was in the range of 5.05–39.63 MPa, depending on age and gender, with the mean value of 16.92 ± 8.76 MPa. These values have the same order of magnitude as those previously reported in other studies. For example, the elastic modulus of human tracheal cartilage has been previously reported to be in the range of 2.5 to 7.7 MPa [22], 1.8 to 15 MPa [17], 1 to 20 MPa [4], or almost 3.3 MPa [14]. The existing difference between our results and those of other studies is partly related to the differences in the number and age of subjects under study, aside from some differences in the applied methods.

Both trachealis muscle and connective tissue exhibited the well-known “stiffening” behavior, which is usually observed in the case of many soft biological tissues. Here, making the isotropy assumption, such nonlinear behavior of both tissues was best described by the Yeoh and Mooney-Rivlin models. Making the isotropy and hyperelasticity assumptions, trachealis muscle and connective tissue had been previously described by Ogden [16], Marlow [2], and Mooney-Rivlin [15] strain energy functions.

In the present study, we assumed all the three tracheal components as isotropic materials, an assumption that simplified the constitutive equations and reduced the number of mechanical tests required for the relatively large population of samples under study. However, different soft tissues of trachea have been previously reported to be anisotropic, exhibiting different stress-stretch behaviors in different directions. For instance, in the circumferential direction, smooth muscle displayed a higher extensibility, and the adventitia membrane exhibited a higher stiffness [3]. In addition, in a former study trachealis muscle was reported to be stiffer in the longitudinal direction [24].

Moreover, we made a comparison in terms of mechanical properties of three tracheal tissues with respect to age, as well as the gender, of the samples. Results were generally indicative of trachea stiffening with aging (see Figure 4). This was most evident in the case of tracheal cartilage. Assuming a linear elastic behavior (until stretch of 1.2), we noticed a significant increase in the stiffness of this tissue with aging (*p* < 0.05), in agreement with the previously reported increases in cartilage tensile stiffness with age [4,17]. At stretch levels greater than 1.2, the same age-related behavior could be observed.

Some alterations in the microstructure of tracheal cartilage with aging have been previously reported, implying that changes in the charge density and size of proteoglycan and aggregation properties along with alterations in their distribution may be influential on the observed age-related changes in the biomechanics of tracheal cartilage [25]. In the present work, we observed cartilage ossification with aging, which can explain the observed stiffer behavior of older cartilage samples. This observation is in agreement with the findings of a previous work reporting the ossification of human tracheal cartilage in aged samples [26].

As is evident in Figure 4, the age-related stiffening was also noticeable in the case of connective tissue. We observed the formation of fibrosis tissue with aging, which may play a part in the observed age-related stiffening of the connective tissue.

Regarding tracheal smooth muscle, it has been previously reported that the static stiffness of this tissue is not age-dependent [27]. In another study on two old and one young subjects, although no age-related comparison had been made and the results were limited by the low number of subjects, comparison of the stress-stretch curves was indicative of a relatively stiffer behavior of trachealis muscle in old subjects compared to the young sample [3]. In the present study, there was a slight difference between the two groups of age, and the old samples of trachealis muscle were slightly, but not significantly stiffer (*p* > 0.05).

In a few studies, the effect of gender on some airway characteristics has been investigated, the results of which were indicative of no dependency of trachea resistance and collapsibility on gender [28], or some effects of such factor on the measured parameters of respiration [29]. In the present study, no significant difference in the mechanical properties of female and male tracheal components was observed. However, cartilage stiffening with aging is stronger in the case of male subjects, compared to females, as opposed to connective tissue which stiffens more with aging in females compared to males. This observation may be related either to the effect of gender or may arise from differences in lifestyle and habits, such as smoking among female and male subjects.

The results of mechanical characterization of human tracheal components can be applied in the study of pathogenic and clinical conditions of trachea and may assist in proper treatment strategies, most notably in the design and fabrication of scaffolds for trachea tissue engineering, in which mechanical compatibility is required in addition to biocompatibility.

## 4. Materials and Methods

In the present study, to evaluate mechanical properties of different tracheal components, three types of tracheal tissues were subjected to uniaxial tensile testing and the obtained data were fitted by several hyperelastic models to select the best-fitting model for each tissue. To do this, after the isolation of trachea from brain-dead subjects and storage in standard conditions, samples of tracheal cartilage, smooth muscle, and connective tissue were prepared and subjected to uniaxial tension to obtain force-displacement curves and then Cauchy stress-stretch data were calculated accordingly. Several strain energy functions were used to fit the experimental data so as to obtain the best-fitting hyperelastic function to mechanically describe each tissue. The mechanical properties were also compared in terms of age and gender (of the 13 young samples, five were from female subjects and eight were from male subjects; and 17 old samples, seven were from female subjects and 10 were from male subjects). Young subjects were 18 to 36 years old with an average age of 26.54 ± 6.45 years, while old subjects were 49 to 65 years old with an average age of 55.88 ± 6.29 years. Observed differences between the mechanical properties of young and old samples were related to some histological alterations in the tissues with aging. For each trachea, three samples of each tracheal tissue (*i.e.*, cartilage, smooth muscle, and connective tissue) were tested and the results were averaged. All data were expressed as mean ± STD. SPSS 16.0 software (SPSS Inc., Chicago, IL, USA) was used for statistical analysis. First, we examined the normality of data distribution for each tracheal component based on Q-Q plots and numerical analysis, the results of which confirmed the normal distribution of data. Then a Two-way ANOVA analysis was performed for a comparison of the results, in terms of age and gender, assuming a significance level of 0.05.

### 4.1. Sample Preparation

**Figure 7:**
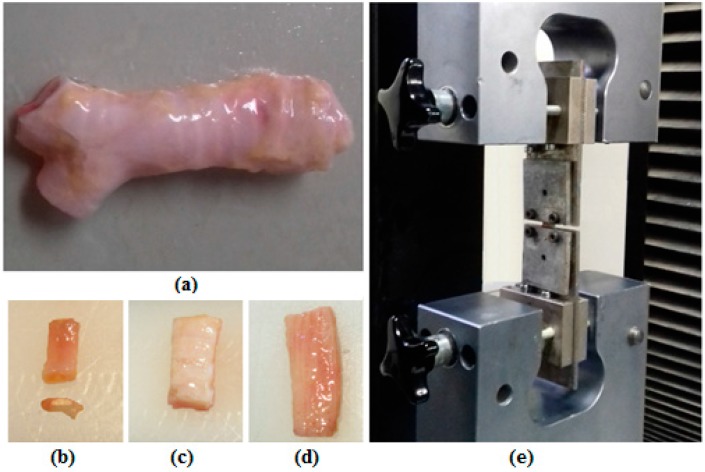
(**a**) A human trachea sample, samples of (**b**) cartilage (along with its cross section); (**c**) connective tissue; (**d**) trachealis muscle; and (**e**) a sample under uniaxial tension.

Human trachea samples of 30 brain-dead human subjects with healthy airways were obtained under the approval of the Ethical Committee of Masih Daneshvari Hospital, as one of the main organ donation sites in Iran (ethics code: sbmu1.rec.1393.73). In order to minimize tissue deterioration after isolation, each sample was immediately immersed in a physiologic saline solution, a suitable preservation solution for trachea samples [30], and then stored at −20 °C, since immediate freezing and then testing right after thawing has been suggested to minimize the risk of postmortem autolysis [14,17,31] and even has been reported to make no significant alteration in the biomechanical properties of the tissue compared to the fresh samples [31]. Trachea samples were transferred to the refrigerator at 4 °C 24 h before performing the experiments, and were put in the ambient temperature 3 h before the tests. Cartilage, smooth muscle, and connective tissue samples were separated with special care and cut into specified dimensions for mechanical testing (Figure 7). To prepare cartilage samples, only the straight central part of the cartilaginous ring was used so as to avoid the ring curvature [14]. In the case of connective tissue, based on a previously proposed method [2], we tested composite samples of consecutive cartilage and connective tissue layers. Assuming the stiff cartilaginous parts as rigid regions of the sample, we considered deformation to only occur in the connective tissue region. To measure the net L_0_ for this tissue, the image taken before starting each test (after reaching a preload of 0.1 N) was used to precisely determine the dimensions of connective tissue using AutoCAD 2009 software (Autodesk, San Rafael, CA, USA). The displacement data recorded by the tensile machine, in association with the final measured L_0_, were used to calculate stretch using the testXpert II v3.0 software (Zwick Roell Group, Ulm, Germany). The average lengths (L_0_) corresponding to cartilage, smooth muscle, and connective tissue samples were 4.13 ± 1.81 mm, 6.37 ± 1.91, and 1.60 ± 0.33 mm, respectively.

### 4.2. Mechanical Tests

Uniaxial tensile tests were carried out at room temperature on samples of cartilage, smooth muscle (connecting the ends of cartilaginous rings), and connective tissue (connecting consecutive cartilaginous rings) using a Zwick/Roell testing machine (with a 2 kN KAP-TC load cell with a least sensitivity of 0.0001 N, Zwick Roell group, Ulm, Germany). In order to enhance the gripping and prevent specimen sliding during mechanical tests, sandpaper pieces were attached to the grips of the testing machine.

For each sample, first a preconditioning phase was performed to achieve repeatable results [2]. Then uniaxial tensile tests were carried out while samples were moisturized with a physiological saline solution during the tests. In order to test each cartilage sample, it was first preconditioned using six loading/unloading cycles (0.1 mm/min strain rate, maximum strain amplitude of 1%) and then underwent uniaxial tension with a strain rate of 0.5 mm/min until failure of the sample. To test connective tissue and smooth muscle samples, each specimen was subjected to six loading/unloading cycles (3 mm/min strain rate, maximum strain amplitude of 5%) followed by uniaxial tension, with a strain rate of 1 mm/min until failure of the sample. Preconditioning and testing parameters were chosen based on a previously published protocol [19] for the characterization of tracheal components. The data were recorded as force *vs.* displacement, and then Cauchy stress *vs.* stretch data were calculated using the following formulations, assuming samples as incompressible materials (Equation (1)):

(1) $$A_{0}L_{0}=A_{t}L_{t}=constant$$

(2) $$\lambda =\frac{L_{0}+∆L}{L_{0}}$$

(3) $$\sigma =\frac{T}{A_{0}}\lambda$$

Here, *L*_0_ and *A*_0_ are the initial length and cross section area of the sample, and *L* and *A* denote the length and cross section area of the sample at any time point t during the test, respectively. Parameters *T* and ∆*L* indicate the tensile force and displacement values recorded during the test and finally, *λ* and *σ* are stretch and Cauchy stress, respectively.

### 4.3. Hyperelastic Modelling of Tracheal Components

The three tracheal components (*i.e.*, cartilage, smooth muscle, and connective tissue) were considered as isotropic and incompressible hyperelastic tissues [2]. The average stress-stretch curve corresponding to each component was fitted to several strain energy potential functions using the curve fitting toolbox in Matlab software, based on the Levenberg-Marquardt algorithm. The best-fitted hyperelastic model for each component was used to describe its nonlinear elastic behavior.

For an isotropic hyperelastic material, the strain energy function depends on strain invariants:

(4) $$W_{isotropic}=W\left(I_{1},\;I_{2},I_{3}\right)$$

In which:

(5) $$I_{1}=\lambda_{1}^{2}+\lambda_{2}^{2}+\lambda_{3}^{2}\;I_{2}=\lambda_{1}^{2}\lambda_{2}^{2}+\lambda_{2}^{2}\lambda_{3}^{2}+\lambda_{1}^{2}\lambda_{3}^{2}\;I_{3}=\lambda_{1}^{2}\lambda_{2}^{2}\lambda_{3}^{2}$$

where *λ*_1_, *λ*_2_ and *λ*_3_ are principal stretches. Therefore, the strain energy function can be written as a function of stretches:

(6) $$W_{isotropic}=W\left(\;\lambda_{1},\;\lambda_{2},\lambda_{3}\right)$$

The deformation gradient tensor (*F*) is used to describe a finite deformation:

(7) $$F_{i,j}=\frac{\partial x_{i}}{\partial X_{j}};\;i,j=1,2,3$$

In which, *X* and *x* indicate the point coordinates in reference and deformed configurations. In the case of uniaxial tension in the x_1_ direction, considering *λ*_1_ = *λ* and imposing the compressibility condition (J = det (*F*) = 1) which results in *λ*_2_ = *λ*_3_ = 1/$\sqrt{\lambda }$, F can be written as:

(8) $$\left[F\right]=\left[\begin{array}{ccc} \lambda & 0 & 0\\ 0 & \frac{1}{\sqrt{\lambda }} & 0\\ 0 & 0 & \frac{1}{\sqrt{\lambda }} \end{array}\right]$$

The right and left Cauchy-Green tensors ([*c*] and [*b*], respectively) can be obtained based on deformation gradient using the following formulations:

(9) $$\left[c\right]={\left[F\right]}^{T}\left[F\right]\;,\;\left[b\right]=\left[F\right]{\left[F\right]}^{T}$$

and using Equation (8) they are calculated to be equal:

(10) $$\left[c\right]=\left[b\right]=\left[\begin{array}{ccc} \lambda^{2} & 0 & 0\\ 0 & \lambda^{-1} & 0\\ 0 & 0 & \lambda^{-1} \end{array}\right]$$

The invariants can be determined using the right Cauchy tensor (Equation (10)):

(11) $$I_{1}=tr\left(c\right),\;I_{2}=\frac{1}{2}(\left(tr\left(c\right){)}^{2}-tr\left(c^{2}\right)\right),\;I_{3}=\det\left(c\right)$$

Therefore in this case, the invariants can be calculated as:

(12) $$I_{1}=\lambda^{2}+\frac{2}{\lambda }\;,\;I_{2}=2\lambda +\frac{1}{\lambda^{2}}\;,\;I_{3}=1$$

Regarding the principal Cauchy stresses for incompressible, isotropic, and hyperelastic materials under uniaxial tension, *σ*_2_ = *σ*_3_ = 0 and *σ*_1_ can be written based on invariants or stretches [32]:

(13) $$\sigma_{1}=\lambda_{1}\frac{\partial W}{\partial \lambda_{1}}-\lambda_{3}\frac{\partial W}{\partial \lambda_{3}}$$

(14) $$\sigma_{1}=2\left(\lambda^{2}-\frac{1}{\lambda }\right)\left(\frac{\partial W}{\partial I_{1}}+\frac{1}{\lambda }\frac{\partial W}{\partial I_{2}}\right)$$

Here, W is the strain energy function. Different expressions for strain energy functions have been previously proposed, based on statistical mechanics, or invariant- or stretch-based continuum mechanics [33]. The utilized hyperelastic models in the present study, namely Mooney-Rivlin, Yeoh, Fung, Neo-Hookean, Humphrey, Ogden, and Veronda–Westmann (see [33] for a full review) along with their corresponding Cauchy stress formulations obtained from Equations (13) and (14) for incompressible and isotropic materials under uniaxial tension [32] are listed in Table 3.

### 4.4. Histological Staining

Sample cuts of human tracheal components (*i.e.*, cartilage, smooth muscle, and connective tissue) of young and old subjects without any tracheal disorders were used for histological analysis according to a previously published protocol [14]. Briefly, samples were fixed in formalin 10% for 24 h at room temperature and then transferred to alcohol (70 °C). They were embedded and sectioned in 5 μm-thickness slices and then stained with haematoxylin and eosin (HE), as well as Masson’s trichrome stains. Samples were observed under a light microscope (BX51, Olympus Corporation, Tokyo, Japan).

## 5. Conclusions

In the present study, we evaluated the mechanical properties of the main constituents of human trachea, including cartilage, connective tissue and smooth muscle using uniaxial tensile tests. Among different hyperelastic models, Yeoh and Mooney-Rivlin models were best able to describe the behavior of all tracheal components. We also compared the results, in terms of age and gender. While no significant effect of gender on the results was observed, we noticed a general stiffening of human trachea with aging (which was most evident in the case of cartilage). Consistently, we found some histological changes in the tracheal cartilage and connective tissue, with aging. The results of this study can enhance our understanding of the mechanics of human trachea.

## Figures and Tables

**Table 1:** Fitting parameter, *R*^2^, for fitting different hyperelastic models to the three tracheal components.

Model	Cartilage	Smooth Muscle	Connective Tissue
Mooney-Rivlin	0.999	0.999	0.999
Yeoh	0.999	1.000	1.000
Fung	0.999	0.988	0.995
Neo-Hookean	0.999	0.871	0.923
Humphrey	0.999	0.988	0.995
Ogden	0.999	0.927	0.968
Veronda-Westmann	0.994	0.993	0.998

**Table 2:** Coefficients of Yeoh and Mooney-Rivlin hyperelastic models for the three tracheal components.

Model	Tissue	*R*^2^	a_1_	a_2_	a_3_
	Cartilage	0.999	3.583	−2.534	12.020
Yeoh	Smooth Muscle	1.000	0.063	0.394	−0.171
	Connective Tissue	1.000	0.257	0.483	−0.148
	Cartilage	0.999	2.766	0.770	-
Mooney-Rivlin	Smooth Muscle	0.999	1.164	−1.223	-
	Connective Tissue	0.999	1.836	−1.775	-

**Table 3:** Utilized strain energy functions and their corresponding Cauchy stress expressions for an incompressible isotropic material under uniaxial tension.

Model	Strain Energy Function	Cauchy Stress
Mooney-Rivlin	$W=\overset{2}{\underset{i=1}{\sum }}a_{i}\left(I_{i}-3\right)$	$\sigma =\left(\lambda^{2}-\frac{1}{\lambda }\right)(a_{1}+\frac{a_{2}}{\lambda }$*)*
Yeoh	$W=\overset{3}{\underset{i=1}{\sum }}a_{i}{\left(I_{1}-3\right)}^{i}$	$\sigma =a_{1}\left(I_{1}-3\right)+a_{2}{\left(I_{1}-3\right)}^{2}+a_{3}{\left(I_{1}-3\right)}^{3}$
Fung	$W=\frac{a_{1}}{2a_{2}}\left(e^{a_{2}\left(I_{1}-3\right)}-1\right)$	$\sigma =a_{1}\left(\lambda^{2}-\frac{1}{\lambda }\right)e^{a_{2}\left(I_{1}-3\right)}$
Neo-Hookean	$W=a_{1}\left(I_{1}-3\right)$	$\sigma =2a_{1}\left(\lambda^{2}-\frac{1}{\lambda }\right)$
Humphrey	$W=a_{1}\left(e^{a_{2}\left(I_{1}-3\right)}-1\right)$	$\sigma =2a_{1}a_{2}\left(\lambda^{2}-\frac{1}{\lambda }\right)e^{a_{2}\left(I_{1}-3\right)}$
Ogden	$W=\overset{3}{\underset{n=1}{\sum }}\frac{a_{\left(2n-1\right)}}{a_{2n}}(\lambda_{1}^{a_{2n}}+\lambda_{2}^{a_{2n}}+\lambda_{3}^{a_{2n}}-3)$	$\sigma =a_{1}\left(\lambda^{a_{2}}-2^{a_{2}-1}\lambda^{-a_{2}/2}\right)+a_{3}\left(\lambda^{a_{4}}-2^{a_{4}-1}\lambda^{-a_{4}/2}\right)+a_{5}\left(\lambda^{a_{6}}-2^{a_{6}-1}\lambda^{-a_{6}/2}\right)$
Veronda–Westmann	$W=a_{1}\left(e^{a_{2}\left(I_{1}-3\right)}-1\right)-\frac{a_{1}a_{2}}{2}\left(I_{2}-3\right)$	$\sigma =2a_{1}a_{2}\left(\lambda^{2}-\frac{1}{\lambda }\right)\left(e^{a_{2}\left(I_{1}-3\right)}-\frac{1}{2\lambda }\right)$
